# Studying Cannabis Use Behaviors With Facebook and Web Surveys: Methods and Insights

**DOI:** 10.2196/publichealth.9408

**Published:** 2018-05-02

**Authors:** Jacob T Borodovsky, Lisa A Marsch, Alan J Budney

**Affiliations:** ^1^ The Center for Technology and Behavioral Health Dartmouth Geisel School of Medicine Lebanon, NH United States

**Keywords:** epidemiology, cross-sectional studies, sampling studies, social media, data collection, cannabis, surveys

## Abstract

The rapid and wide-reaching expansion of internet access and digital technologies offers epidemiologists numerous opportunities to study health behaviors. One particularly promising new data collection strategy is the use of Facebook’s advertising platform in conjunction with Web-based surveys. Our research team at the Center for Technology and Behavioral Health has used this quick and cost-efficient method to recruit large samples and address unique scientific questions related to cannabis use. In conducting this research, we have gleaned several insights for using this sampling method effectively and have begun to document the characteristics of the resulting data. We believe this information could be useful to other researchers attempting to study cannabis use or, potentially, other health behaviors. The first aim of this paper is to describe case examples of procedures for using Facebook as a survey sampling method for studying cannabis use. We then present several distinctive features of the data produced using this method. Finally, we discuss the utility of this sampling method for addressing specific types of epidemiological research questions. Overall, we believe that sampling with Facebook advertisements and Web surveys is best conceptualized as a targeted, nonprobability-based method for oversampling cannabis users across the United States.

## Introduction

Surveys have been a methodological cornerstone of epidemiology since the inception of the field. However, the manner in which epidemiologists conduct surveys has undergone several paradigm shifts in tandem with advances in mass communication and information dissemination [1]. Initially, data collection was limited to local in-person site visits [1,2]. Over time, new modes of communication, such as mailed questionnaires and random digital dialing [3], expanded epidemiologists’ methodological toolkit [1]. Once the internet began to function as a principal means of communication, it too was recognized for its potential utility as a data collection method [1,4,5]—particularly for substance use data [6]. Historically, collecting data on hidden and stigmatized populations such as substance users had been exceptionally difficult. Early pioneering work demonstrated that it was possible to use the internet to study these populations [7,8].

Today, approximately 3.5 billion people around the world have internet access [9], and 2.3 billion people own a smartphone [10], allowing them to access the internet at any time of day in almost any location. Approximately 2.5 billion people [11] now use social media and networking sites such as Facebook and Twitter, primarily on their smartphones [12,13], for an average of 1 to 2 hours per day [14,15]. In parallel with this increase in internet and social media use, researchers have devoted considerable attention to conducting Web-based studies of health behaviors. In doing so, they have developed various Web survey- and social media-based data collection methods [16-24]. Of the social media platforms now commonly used for health research purposes, Facebook, in particular, has emerged as a useful and low-cost means of recruiting participants [25] from hard-to-reach populations [16]. At present, researchers have used Facebook to disseminate Web surveys to study a range of diseases and health behaviors such as HIV [26]; vaccine uptake [27,28]; mammographies [29]; contraception [30]; several mental health disorders [31-33]; prescription medication misuse [34]; and use of alcohol [35], tobacco [36], e-cigarettes [37], and cannabis [38-42]. Such studies are part of a growing subdiscipline of epidemiology—often termed *Infodemiology* [43], *Digital Epidemiology* [44], or *E-Epidemiology* [45]—that is characterized by the overlap between traditional epidemiological research goals and the utilization of new digital infrastructures.

Facebook-based Web surveys are well suited for addressing questions that arise from sociocultural changes because they allow for the rapid study of behaviors on a population-level scale. One example of such a sociocultural change is the shifting legal landscape of cannabis in the United States. More recreational and medical legal cannabis laws (LCL) have been enacted since 2010 than were enacted from 1996 to 2009 (LCL is used here to differentiate such laws from those focused on criminalizing and prohibiting cannabis) [46], and several new concerns—including the expansion of cannabis product diversity [47-50]—have emerged as a result. Many of the questions brought about by cannabis legalization represent excellent scientific targets for Facebook-based sampling methods.

At the Center for Technology and Behavioral Health at Dartmouth College, we have been leveraging the Facebook Business advertising platform to conduct a series of Web surveys to study questions concerning patterns of use of new methods of cannabis administration (eg, vaping and edibles) and relationships between cannabis use and psychological constructs such as anxiety, pain, and readiness to reduce or stop cannabis use. In the process of conducting these surveys, we have learned several lessons about advertising procedures that minimize costs and maximize survey participation. In addition, given the need to determine strengths and limitations of social media-based data collection [51], we have aggregated and examined the data from several of our surveys to identify the unique characteristics of our sampling method.

This paper provides an overview of our procedures and lessons learned using Facebook advertisements and Web surveys as a method to study cannabis use, our findings regarding the unique characteristics of the cannabis use data produced by this method, and how the characteristics of the resulting data clarify the types of research questions best suited for study with this sampling method.

## Using the Facebook Advertising Platform

### Targeting Parameters

The Facebook advertising platform provides researchers with access to a large sample pool and a wide range of demographic, behavioral, and psychographic targeting parameters. These parameters can be tailored to send customized advertisements (eg, recruitment messages for a Web survey) to the phone and computer screens of specific populations of interest [31,52,53]. This sampling capability is possible because of the vast amount of information Facebook collects about each user’s Web-based behavior.

General targeting parameter categories include any age range above 13 years, gender, education (eg, type of educational degree), relationship status (eg, marital status), financial status (eg, income level), geographic location, multicultural affinity (eg, African American, Hispanic, Asian), generation (eg, baby boomers), employment (eg, job title), household composition (eg, new parents), and Web-based purchase behaviors (eg, pain relief medications, alcoholic beverages).

Our research group has been interested in studying cannabis use. However, Facebook does not provide targeting parameter categories such as *cannabis user*. Thus, in our advertising strategy, we use targeting parameters that we believe are correlated with our behavior of interest (cannabis use), such as notable individuals associated with cannabis use (eg, Bob Marley, Ed Rosenthal), cannabis-related magazines (eg, Cannabis Culture, High Times), organizations (eg, Americans for Safe Access, NORML, Weedmaps), and behaviors or topics (eg, “smoking weed”,“legalize marijuana”).

### Algorithm Learning and Optimization

**Figure 1:**
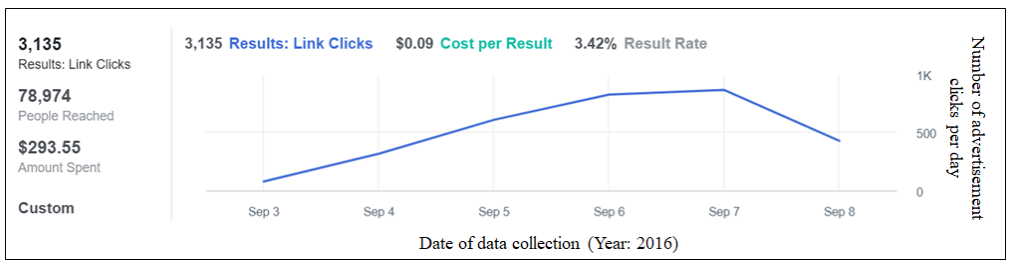
Click and cost results from a recent cannabis-targeted Facebook advertising campaign.

Facebook’s machine learning–based algorithms are designed to present users with content (including advertisements) relevant to their personality or lifestyle [54-56]. However, the algorithms used to distribute advertisements appear to require a sufficient amount of time to complete a learning phase before they become effective. During this learning phase, enough data must be accumulated to determine which members of the target population have the highest probability of engaging with the advertisement [54-56]. Although we cannot verify that such learning processes are taking place or how they are programmed, our experiences using the Facebook advertisement system to date have been congruent with this literature. Currently, when we use Facebook advertisements to reach cannabis users, we begin advertising with a low spending limit of US $10 per day for 48 to 72 hours, which we believe facilitates algorithm learning. After this 48- to 72-hour time frame, we have consistently seen a notable increase in the rate of clicks. At that point, we have increased the amount of money spent to anywhere between US $30 and US $60 per day. Figure 1 displays the Facebook advertisement click results from our most recently published survey [57]. In this study, we preprogrammed advertisements to be displayed for 6 days (September 3-8, 2016). As is evident from Figure 1, we received few advertisement clicks during the first 24 hours of advertising (September 3). However, after 72 hours, the advertisement was receiving over 500 clicks per day. Of note, Facebook paces advertisement spending to remain within-budget over the course of the entire advertising time frame [58]. We believe this is why the number of clicks per day begins to decline toward the end of the advertising time frame (ie, September 8 in Figure 1).

It is important to note that this specific 48- to 72-hour time frame may not apply to other social media advertising platforms or even to Facebook in the future. However, we believe that the principle for effectively using machine learning–based advertising demonstrated here will still hold.

**Figure 2:**
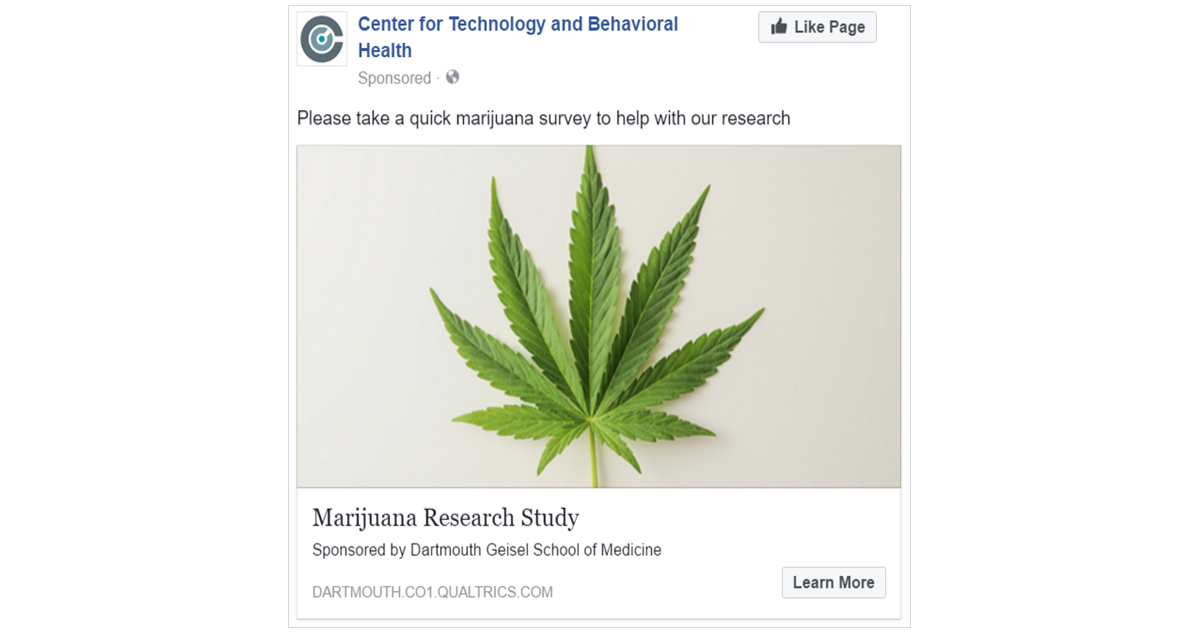
Sample advertisement distributed via Facebook advertising.

### Advertisement Imagery and Data Collection

To sample cannabis users in these studies, we direct Facebook recruitment advertisements (Figure 2) to individuals who live in the United States and are affiliated with cannabis-related targeting parameters discussed previously. In the advertisements, we use images and wording that are salient to the population we want to study. This means our advertisements frequently display cannabis leaves and use the words *cannabis* or *marijuana* in the text. The advertisements contain the URL link to our anonymous Qualtrics-hosted survey. Individuals who click the advertisement are redirected to the consent page of the survey. Individuals are excluded if they (1) do not provide consent, (2) do not meet study-specific age requirements (eg, aged above 18 years), or (3) self-report never having used cannabis. We use internal data checks to confirm the veracity of the data. For example, individuals who report their age of initiation of cannabis as being older than their current age are excluded from analyses. We disable internet protocol (IP) address collection to maintain participant anonymity and describe this procedure and its implications clearly on the consent page. We enable Qualtrics data quality features that use cookies to prevent individuals from responding multiple times. We also use the captcha verification feature to prevent responses from internet bots. We have not used any survey completion compensation or incentives in these studies.

### Iterative Questionnaire Modifications

In addition to using the sampling strategies discussed previously, we have also iteratively modified the structure of our questionnaires in several ways. Note that our team has not attempted to evaluate the isolated impact of each of these modifications systematically, and thus we cannot comment on causal relationships. However, based on our experiences to date, we believe that the following questionnaire modifications have helped increase the likelihood of participation and completion: (1) using language at the top of the consent page that highlights the changing landscape of US cannabis legalization and the need for public contribution to better understand cannabis; (2) using the term *cannabis* rather than *marijuana*; (3) using patently objective language about cannabis on the consent page (ie, explaining that our research team’s primary aim is to collect accurate data—not promote or demonize cannabis); (4) conveying to participants, at the top of the consent page, that the questionnaire will only take 10 to 15 min to complete; (5) conveying to participants that their IP addresses will not be tracked; (6) shortening the overall length of the questionnaire; (7) using images of cannabis and methods of cannabis administration throughout the questionnaire; (8) ensuring that the first few questions that participants see immediately after providing consent are interesting to them (ie, “Have you ever used cannabis?”); (9) distributing uninteresting questions (eg, demographic questions) throughout the questionnaire; and (10) providing an open-ended, free-response item that asks participants for their thoughts about both positive and negative experiences with cannabis.

We believe that our surveys have become increasingly well received by the Facebook community as we have made these changes over time. For example, in our second survey—conducted over 28 days for US $809—our advertisements received 107 *likes* (ie, a positive reaction to the advertisement), 32 comments, and 27 *shares* (ie, an individual sending the advertisement to one of their friends via Facebook). In our fourth survey—conducted over 6 days for US $293—we received 354 likes, 41 comments, and 139 shares. Content analysis of the reactions and comments to the various surveys over time is beyond the scope of this paper, but we believe that the patterns observed generally show increased acceptance and willingness to engage and participate in these types of survey studies.

## Cannabis-Related Findings From Facebook Advertising–Generated Survey Data

We have conducted a series of Web surveys using these methods, which have provided insights into patterns of cannabis use across the United States. Below we present data from 6 of those surveys, 3 of which have been published [57,59,60]. Table 1 provides an overview of the recruitment results for each of the surveys.

In our initial study, we used Facebook advertising to recruit adults (N=2910) for a survey on cannabis vaping and edible use. We found that over half (61.27%, 1783/2910) of the users had tried vaping, but only 12.44% (362/2910) of the sample preferred vaping over other methods [61], and the likelihood of vaping and edible use was positively associated with the number of within-state cannabis dispensaries per capita [59]. A second study replicated these findings with cannabis users (N=933). Again, the majority (55.2%, 515/933) had tried vaping, and of those, 27.2% (140/515) had vaped while driving, 34.2% (176/515) had vaped in public, and 14.2% (73/515) had vaped at work (A J Budney, unpublished data, July 2016). In a third study, we collected cannabis use survey data from younger cannabis users (aged 14-18 years; N=2630) and replicated our previous adult findings. We observed the same relationship between dispensaries and vaping and edible use. We also found that home cultivation provisions of legal cannabis laws were uniquely related to a greater likelihood of having used edibles [60]. In a follow-up survey of adults, we collected responses (N=1813) to explore the relationship between provisions of legal cannabis laws (home cultivation and dispensaries) and cannabis edible procurement behaviors. We determined that those who live in states that permit home cultivation tend to make their own cannabis edibles, whereas those who live in states with cannabis dispensaries primarily purchase their edibles [57]. In another study, cannabis users (N=1212) participated in a survey assessing the frequency of cannabis use and thoughts about decreasing their use. A sizeable portion (19.39%, 235/1212) reported having “been concerned about their cannabis use,” and, among these individuals, 75.32% (177/235) had recently thought about reducing their use (A J Budney, unpublished data, February 2017). In another study, cannabis users (n=3561) participated in a survey on frequency of cannabis use and selected psychological processes. Indirect effects of anxiety sensitivity on past 30-day heavy cannabis use were mediated by coping-related motives for cannabis use (A A Knapp, unpublished data, November 2016).

This Facebook-based Web survey approach can be particularly useful for expeditiously conducting studies that help clarify observations and questions that arise from prior survey studies. For example, the primary analyses of the youth survey discussed previously revealed that state-level permission of cannabis home cultivation was statistically related *only* to an elevated likelihood of edible use, whereas state-level permission of cannabis dispensaries was related to elevated likelihoods of *both* lifetime vaping and edible use. After developing a hypothesis to explain this observation, we conducted a new survey 3 months later to better understand these relationships. These data replicated the results from the prior study and further demonstrated that those who grow cannabis were more likely to make edibles at home by economizing low-tetrahydrocannabinol (THC) leftover parts of the cannabis plant. However, those who lived in states with dispensaries were more likely to purchase cannabis edibles [57].

**Table 1:** Recruitment results from 6 cannabis use Web surveys disseminated using Facebook advertisements (ads).

Survey^a^	Ad delivery time frame	Total ad cost ($ USD)	No. of people who saw ads	No. of ad clicks	Sample size^b^	Ad images	Demographics and cannabis use patterns of recruited sample			
							Age, mean (SD)	Male, n (%)	White, n (%)	Current use^c^, n (%)
Survey 1^d^	43 days	800	168,894	3708	2838	Cannabis leaf; College logo	32 (16)	2391 (84.24)	2048 (72.16)	2333 (82.20)
Survey 2	28 days	809	231,400	3932	933	Multiple^e^	44 (18)	758 (81.2)	794 (85.1)	724 (77.6)
Survey 3	20 days	350	126,945	5480	2630	Cannabis leaf	16 (1)	1201 (45.67)	2067 (78.60)	2185 (83.08)
Survey 4	6 days	293	78,974	3135	1813	Cannabis leaf	48 (13)	1386 (76.48)	1608 (88.70)	1540 (85.94)
Survey 5^d^	9 days	402	68,525	2599	1212	Cannabis leaf	28 (11)	784 (64.69)	1029 (84.90)	1132 (93.40)
Survey 6^d^	7 days	377	96,096	5612	2972	Cannabis leaf; Cannabis plant	35 (10)	1815 (61.07)	2653 (89.27)	2549 (85.77)

^a^Eligibility criteria for all surveys: (1) lifetime cannabis user, (2) age 18 years or older, (3) provided consent/assent, and (4) currently living in the United States. Exception for survey 3 in which the age eligibility criteria was 14 to 18 years.

^b^Respondents who (1) met eligibility criteria, (2) passed data-quality checks, and (3) completed the survey.

^c^“Current use” indicates individuals who used cannabis at least once in the past 30 days.

^d^Participants permitted to skip questions. Reported sample sizes may vary depending on the variable analyzed.

^e^Advertisement images included cannabis leaf, cannabis plant material, Dartmouth College logo, methods of use (eg, joints), depictions of smoking behavior, and smoke clouds.

## Determining and Interpreting the Idiosyncrasies of Facebook Advertising–Based Cannabis Use Data

As this line of research expands, it is crucial to understand both the strengths and limitations of this Facebook survey sampling method. Epidemiologists use a variety of sampling methods to answer different types of research questions [62-65]. For example, accurately estimating the prevalence of lifetime cannabis use in the United States requires probability-based methods, but understanding the unique cannabis use disorder treatment needs of various population subgroups requires nonprobability-based sampling methods [66]. The unique properties of Facebook sampling for studying cannabis use are largely unclear at this point — making it difficult to determine which types of research questions are most effectively addressed with this method. To begin to fill this gap, we have conducted several secondary analyses of our Facebook-based data. On the basis of our understanding that Facebook advertising was originally designed to help businesses contact their specific niche audience, we hypothesized that Facebook advertisements would produce data similar to traditional nonrandom epidemiological sampling methods, thus producing data on a relatively homogenous subpopulation.

### Patterns of Cannabis Use

**Figure 3:**
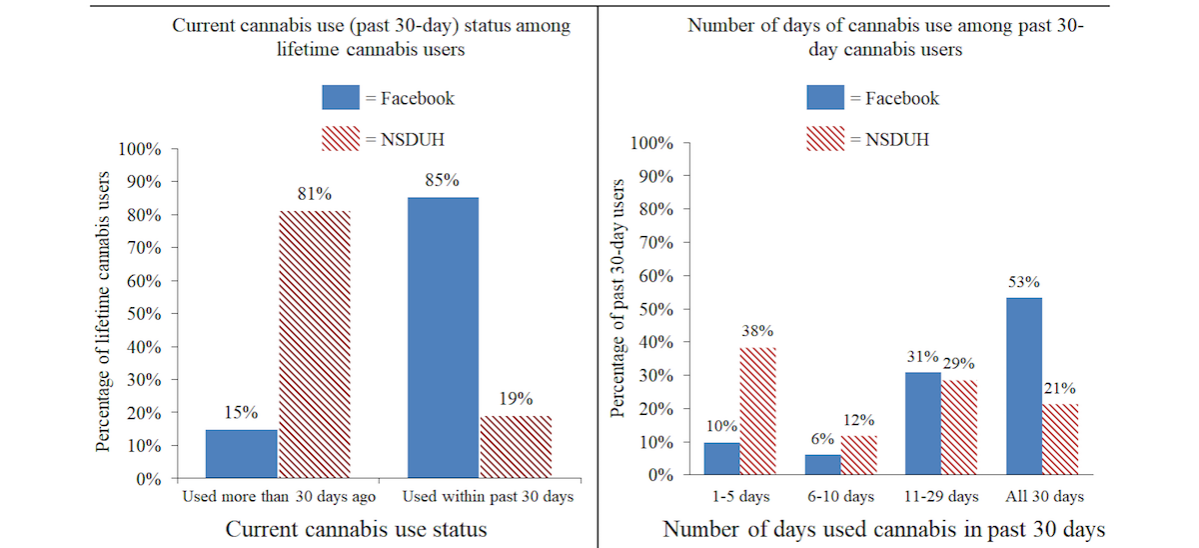
Comparisons between Facebook-generated cannabis use data and cannabis use data from the 2015 National Survey on Drug Use and Health (NSDUH).

In our initial surveys, we were interested in studying patterns of use of novel methods of cannabis administration. We assumed that current and regular cannabis users would be the most likely to have used these methods of administration, and therefore, we attempted to oversample this subgroup by using the cannabis-centric sampling targets (eg, Medical Marijuana or High Times Magazine) discussed previously. To confirm that our sampling method did indeed oversample this subgroup of cannabis users, we compared our data with cannabis use data from the probability-based National Survey on Drug Use and Health (NSDUH). The NSDUH can be used to generate prevalence estimates of multiple subtypes of cannabis users in the United States. Figure 3 displays our Facebook-based data combined across several of our published and unpublished adult (aged above 18 years) surveys (N=10,427; includes individuals who did not complete an entire survey) in relation to data from the 2015 NSDUH [67]. The left panel of Figure 3 shows that samples of lifetime cannabis users from our surveys comprised proportionally more current (ie, used at least one time in the past 30 days) users compared with lifetime users in the NSDUH. In addition, the right panel in Figure 3 shows that our samples of current users (n=8886) comprised proportionally more daily cannabis users compared with current users in the NSDUH [59,60,68]. These findings support the notion that Facebook sampling can effectively obtain data from subgroups of lifetime cannabis users who are currently using cannabis on a regular basis.

Similar comparative analyses by other researchers support this finding. For example, Barratt et al analyzed cannabis use data from the Global Drug Survey (a self-selected Web survey) in relation to several national probability-based surveys (including NSDUH). Their results demonstrate that Web-based purposive recruitment (including the use of Facebook) can generate samples in which current cannabis users are over-represented [69].

### Geographic Representation

**Figure 4:**
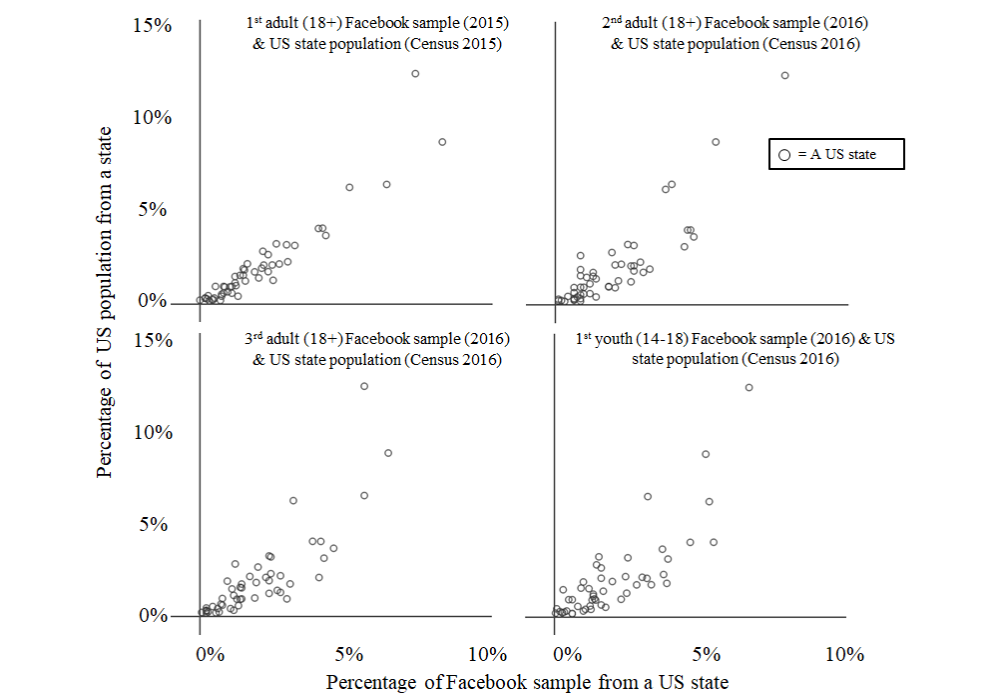
Percentage of US population in each state versus percentage of Facebook sample in each state.

A second consideration is the possibility of a selection bias induced by state residence. Individuals living in medical or recreational LCL states may have a greater (or lesser) propensity to take our surveys than individuals from states that have not legalized cannabis use. To determine if this was occurring, we compared several of our Facebook-based datasets (both published and unpublished) with US census data [70]. As demonstrated in Figure 4, Facebook consistently generates samples in which the proportion of survey respondents from each US state matches the proportion of the total US population represented in each US state. Pearson and Spearman correlation coefficients for each relationship displayed in Figure 4 range from .82 to .95 (*P*<.001). Thus, the results from these data are not necessarily biased by disproportionate geographic representation.

These two characteristics of cannabis use data collected using Facebook advertisements and Web surveys provide some indication as to the types of research questions this method can be useful for studying. Using targeted interests (eg, Medical Marijuana) to recruit participants introduces a selection bias, and our comparisons to NSDUH data suggest that this selection bias generates samples that over-represent current and regular cannabis users. However, our comparisons to the US census indicate that these data are not provincial—Facebook samples individuals from across the United States. Thus, this sampling method seems better suited for research questions aimed at understanding how cannabis legalization may affect current and frequent cannabis users rather than how it will affect inexperienced or infrequent cannabis users.

A strength of this sampling method is that researchers can collect enough data to address their research question after only a few days of data collection. This advantage, however, begs questions related to the potential impact of natural temporal variability on the data and results. For example, how similar are data sampled at the beginning of the week to data sampled at the end of the week? Without answers to this type of question, it is difficult to know the extent to which we should be concerned about temporally related confounding. In our analyses, we have begun preliminary exploration of potential fluctuations in the types of cannabis users who take our surveys on particular days of the week. Table 2 displays demographic and cannabis use characteristics of participants from our recently published youth dataset [60] aggregated by the day of the week that the data were sampled. The resulting profile is relatively stable across days of the week for many variables, but significant variability does occur. Looking at the gender variable in Table 2, it is clear that the distribution of responses across categories within a variable can change multiple times during the week. In addition, abrupt changes in the absolute difference in proportions between categories of a variable can occur (eg, the difference between male and female representation on Saturdays). Given that the sampling strategy remained the same for the duration of the study, it is unclear why such variability occurred and why some variables—such as gender in this particular instance—might be affected more than others. Due to the current degree of uncertainty surrounding this methodological issue, we suggest collecting data for at least a full week to capture any potential temporally related variability in the data. We also suggest that relationships among sample size, effect size, number of statistical tests conducted, and *P* values be considered as a whole to determine whether the observed fluctuations warrant statistical adjustment.

### Additional Considerations for Facebook Sampling

Several other methodological considerations warrant comment. Like other sampling methods, Facebook advertising is subject to both noncoverage and nonresponse biases [71]. There are cannabis users living in the United States who do not have Facebook or have Facebook but do not engage in cannabis-related activity on Facebook (and thus cannot be targeted by our advertising). Other cannabis users may not wish to engage in research studies even if they are presented with the opportunity to do so (ie, they see the advertisement on their screen but do not click it). Although it is difficult to test for systematic differences between those who do and do not click on an advertisement when given a chance, the cannabis users who do click advertisements and take surveys provide generally reliable and diagnostically valid cannabis use data [72].

**Table 2:** Demographic and cannabis use–related data sampled via Facebook advertising according to the day of the week data were collected.

Variable		Day of the week data were collected (N=2630)							*P* value^a^
		Monday^b^ (n=435)	Tuesday^b^ (n=408)	Wednesday^b^ (n=342)	Thursday^c^ (n=257)	Friday^b^ (n=417)	Saturday^b^ (n=365)	Sunday^b^ (n=406)	
**Education,** **n (%)^d^**									.11
	6th grade	0	0	0	0	1 (0.2)	2 (0.5)	1 (0.2)	
	7th grade	8 (1.8)	7 (1.7)	3 (0.9)	4 (1.6)	6 (1.4)	5 (1.4)	2 (0.5)	
	8th grade	34 (7.8)	31 (7.6)	39 (11.4)	16 (6.2)	48 (11.5)	46 (12.6)	43 (10.6)	
	9th grade	89 (20.5)	75 (18.4)	67 (19.6)	51 (19.8)	84 (20.1)	89 (24.4)	87 (21.4)	
	10th grade	135 (31.0)	112 (27.5)	102 (29.8)	73 (28.4)	107 (25.7)	100 (27.4)	109 (26.8)	
	11th grade	108 (24.8)	108 (26.5)	68 (19.9)	71 (27.6)	111 (26.6)	81 (22.2)	110 (27.1)	
	12th grade	40 (9.2)	50 (12.3)	41 (12.0)	27 (10.5)	45 (10.8)	32 (8.8)	44 (10.8)	
	Started college	21 (4.8)	25 (6.1)	22 (6.4)	15 (5.8)	15 (3.6)	10 (2.7)	10 (2.5)	
**Race, n (%)^d^**									.38
	African American	24 (5.5)	12 (2.9)	12 (3.5)	8 (3.1)	9 (2.2)	11 (3.0)	13 (3.2)	
	Native American	13 (3.0)	15 (3.7)	7 (2.0)	9 (3.5)	16 (3.8)	11 (3.0)	7 (1.7)	
	Asian	3 (0.7)	5 (1.2)	5 (1.5)	4 (1.6)	7 (1.7)	3 (0.8)	5 (1.2)	
	White	332 (76.3)	311 (76.2)	274 (80.1)	209 (81.3)	341 (81.8)	281 (77.0)	319 (78.6)	
	Pacific Islander	2 (0.5)	3 (0.7)	0 (0.0)	1 (0.4)	1 (0.2)	1 (0.3)	1 (0.2)	
	Hispanic	61 (14.0)	62 (15.2)	44 (12.9)	26 (10.1)	43 (10.3)	58 (15.9)	61 (15.0)	
**Gender, n (%)**									<.001
	Male	182 (41.8)	197 (48.3)	178 (52.0)	150 (58.4)	191 (45.8)	129 (35.3)	174 (42.9)	
	Female	233 (53.6)	200 (49.0)	155 (45.3)	99 (38.5)	209 (50.1)	223 (61.1)	218 (53.7)	
	Other	20 (4.6)	11 (2.7)	9 (2.6)	8 (3.1)	17 (4.1)	13 (3.6)	14 (3.4)	
**Living situation, n (%)**									.29
	Lives with neither parent	49 (11.3)	43 (10.5)	37 (10.8)	29 (11.3)	44 (10.6)	32 (8.8)	35 (8.6)	
	Lives with both parents	185 (42.5)	187 (45.8)	146 (42.7)	129 (50.2)	176 (42.2)	137 (37.5)	181 (44.6)	
	Lives with mother only	153 (35.2)	132 (32.4)	124 (36.3)	74 (28.8)	155 (37.2)	158 (43.3)	147 (36.2)	
	Lives with father only	48 (11.0)	46 (11.3)	35 (10.2)	25 (9.7)	42 (10.1)	38 (10.4)	43 (10.6)	
**Preferred method, n (%)**									.34
	Smoke cannabis	231 (83.4)	236 (81.1)	207 (85.2)	162 (85.3)	231 (83.7)	202 (86.7)	229 (84.8)	
	Vaporize cannabis	22 (7.9)	21 (7.2)	17 (7.0)	17 (8.9)	17 (6.2)	11 (4.7)	11 (4.1)	
	Eat cannabis	24 (8.7)	34 (11.7)	19 (7.8)	11 (5.8)	28 (10.1)	20 (8.6)	30 (11.1)	
**Lifetime days smoked cannabis, n (%)^e^**									.86
	Once	10 (2.3)	10 (2.5)	6 (1.8)	4 (1.6)	10 (2.4)	10 (2.7)	7 (1.7)	
	2-5 days	28 (6.5)	24 (5.9)	24 (7.1)	12 (4.7)	33 (8.0)	26 (7.1)	27 (6.7)	
	6-10 days	15 (3.5)	24 (5.9)	11 (3.3)	17 (6.6)	22 (5.3)	19 (5.2)	26 (6.4)	
	11-30 days	39 (9.0)	43 (10.6)	45 (13.3)	22 (8.6)	49 (11.8)	36 (9.9)	38 (9.4)	
	31-100 days	68 (15.7)	48 (11.8)	43 (12.7)	38 (14.8)	55 (13.3)	51 (14.0)	50 (12.4)	
	101-365 days	88 (20.4)	93 (22.9)	76 (22.5)	66 (25.7)	86 (20.7)	81 (22.3)	101 (25.0)	
	>365 days	184 (42.6)	164 (40.4)	133 (39.3)	98 (38.1)	160 (38.6)	141 (38.7)	155 (38.4)	

^a^Chi-squared tests used to calculate *P* values.

^b^Data collected on the same weekday but during 2 separate weeks. For example, Monday^b^ indicates data collected on Mondays from 2 different weeks.

^c^Data collected on a weekday on a single week. For example, Thursday^b^ indicates data collected on a Thursday from a single week of data collection.

^d^Sixth and 7th grade combined in education variable, and Asian and Pacific Islander combined in race to conduct chi-squared tests.

^e^n=14 respondents never smoked cannabis (ie, n=14 had only ever used an alternative method of administration such as vaping or edible).

## Discussion and Future Directions

The utilization of social media–based recruitment for conducting cannabis epidemiology research has only begun to scratch the surface of its potential. Our experiences in conducting this research have revealed several insights about this method and the resulting data. First, it would appear prudent to operate under the assumption that Facebook advertising algorithms require a sufficient amount of time to learn about a target population to effectively disseminate advertisements. Thus, we have found it useful to wait at least 48 to 72 hours before evaluating the effectiveness of a Facebook advertising campaign. Second, Facebook advertising methods that recruit for Web-based surveys on cannabis use can quickly and inexpensively generate samples of current, regular cannabis users across the United States. Given the speed of data collection, we have also found that this sampling method is useful for timely clarification of hypotheses, via the expeditious conduct of follow-up surveys. Overall, we encourage the conceptualization of Web survey dissemination via Facebook advertising as a new type of nonprobability-based targeted sampling method. Cannabis researchers can include Facebook as part of their armamentarium of sampling methods. It can be considered as an alternative or adjunct to traditional survey methods, perhaps most valuable when trying to study cannabis behaviors not captured by traditional methods. In addition, in light of the difficulties in developing data infrastructures for evaluating the effects of cannabis legalization [73] and literature suggesting that changes to the legal status of cannabis may disproportionately impact the behavior of regular cannabis users [74], Facebook recruitment methods appear to be valuable for collecting policy-relevant data.

Web surveying via Facebook is only one of many emerging epidemiological methods for studying cannabis use. A growing body of literature has demonstrated that digital trace data [44] (ie, records of naturalistic digital behavior and communication) can be used to study cannabis use. For example, Twitter has been used to study new forms of cannabis administration such as dabbing [75,76], vaping [77], and edibles [78,79]; perceptions, attitudes, and normalization of cannabis use [80-82]; and unique communities of cannabis user subgroups and network structures of cannabis dispensaries [83,84]. Data from other Web-based platforms such as Reddit [85], Instagram [86], YouTube [87], and search engines such as Google [88] or Bing [89] are all other potential sources of digital trace data that have been used to study patterns of cannabis use.

Given the sensitive nature of substance use data, the concept of IP address tracking warrants discussion. When designing a survey, researchers must consider the balance between the need to prevent multiple responses and the need for anonymity to obtain valid responses. Without being able to determine who has already taken a survey, researchers run the risk of having single individuals complete a survey multiple times—especially if monetary incentives are used to encourage survey participation [90]. However, failure to provide respondents with anonymity may result in fewer responses or invalid data. In our surveys to date, we have used the prevent ballot box stuffing feature of Qualtrics as a middle ground. This feature uses cookies rather than IP addresses to prevent multiple responses, which allows us to maintain participant anonymity. Even so, the promise of anonymity may not convince individuals involved in illicit behavior to participate [91]. Finally, note that the use of IP addresses for preventing multiple responses can inadvertently prevent individuals who share the same IP address (eg, college dorm, shared house) from completing the survey.

The landscape of social media will continue to evolve in the coming years, and specific social media platforms such as Facebook may not remain as popular as they are today. However, all indications are that digital social networking and mass communication platforms are here to stay and will continue to grow as advertising tools. In 2017, businesses spent approximately 13.5 billion dollars using social media marketing to sell their products and services [92]. Thus, it seems likely that providing an advertising service within digital social networking platforms will remain a staple means of generating revenue for the companies that create such platforms. In addition, as the machine learning techniques used to disseminate digital marketing advertisements continue to become more sophisticated and effective, researchers can leverage such advances to even more efficiently reach and collect data on clinical subpopulations of interest.

There remain many unanswered questions related to this sampling method. Here, we mention just 3, each of which could provide exciting additional research opportunities. First, can these methods be used effectively to study the use of other substances? It is likely that the degree of stigma surrounding a particular substance of interest will affect the utility of the methodology presented here. For example, there are nationally distributed magazines and well-recognized political activist groups devoted specifically to the promotion of cannabis use and cannabis culture. These aspects of cannabis culture are available as specific targeted interests on the Facebook Business advertising platform. In contrast, to our knowledge, there are no regularly published national magazines or political activist groups that promote crystal methamphetamine use and culture. Thus, the targeting strategies for more stigmatized drugs such as crystal methamphetamine may need to be different and will likely require the use of monetary incentives. One suggestion to overcome this issue is to conduct an in-person pilot focus group with regular users of the substance of interest and collect digital or self-report data concerning their Web-based behavior. The resulting data could then be used to generate a more effective Facebook advertising campaign.

Second, will participants provide identifying contact information in a survey to facilitate longitudinal survey follow-up (ie, repeated sampling from the same individual over time)? Previous work by Harris et al has demonstrated that survey respondents recruited via Facebook are willing to provide contact information to facilitate follow-up for longitudinal studies. However, their study aimed to understand patterns of contraception use among young Australian women [30]. It remains to be seen whether something similar can be accomplished with a US-based sample of heavy cannabis users.

Finally, is it possible to use alternative advertising strategies (eg, not using cannabis-related targeted interests) to obtain samples of less experienced or less frequent cannabis users? Preliminary data currently being collected by our team suggest that this can be readily accomplished using different targeting strategies, but that data collection may be somewhat slower and more expensive.

Sampling issues are a primary concern of any epidemiological investigation because they dictate the conclusions that can be drawn from the data [63,64]. Additional methodological evaluations of social media–based sampling will fill essential gaps in our knowledge of how to use the data effectively. Presently, we believe that social media–based Web surveys have tremendous utility for members of the research community and will continue to facilitate our understanding of the evolving nature of cannabis use behaviors.

## Abbreviations

IP: Internet protocol
LCL: legal cannabis laws
NSDUH: National Survey on Drug Use and Health
